# Optimization of Optical Absorption of Colloids of SiO_2_@Au and Fe_3_O_4_@Au Nanoparticles with Constraints

**DOI:** 10.1038/srep35911

**Published:** 2016-10-27

**Authors:** Xiaozheng Xue, Viktor Sukhotskiy, Edward P. Furlani

**Affiliations:** 1 Department of Chemical and Biological Engineering, University at Buffalo, SUNY, Buffalo, NY, US; 2 Department of Electrical Engineering, University at Buffalo, SUNY, Buffalo, NY, US

## Abstract

We study the optical response of monodisperse colloids of core-shell plasmonic nanoparticles and introduce a computational approach to optimize absorption for photothermal applications that require dilute colloids of non-interacting particles with a prescribed volume fraction. Since the volume fraction is held constant, the particle concentration is size-dependent. Optimization is achieved by comparing the absorption spectra of colloids as a function of particle size and structure. We demonstrate the approach via application to colloids of core-shell SiO_2_@Au and Fe_3_O_4_@Au nanoparticles with particle sizes that range from 5–100 nm and with the incident wavelength varying from 600–1200 nm. The absorption spectra are predicted using Mie theory and the analysis shows that there is a unique mix of parameters (core radius, shell thickness, wavelength) that maximize absorption, independent of the value of volume fraction. We show that lossy Fe_3_O_4_ cores produce a much broader absorption peak with much less sensitivity to variations in particle structure and wavelength than lossless SiO_2_ cores. This approach can be readily adapted to colloids of nanoparticles with arbitrary materials, shapes and structure using appropriate numerical methods to compute the absorption spectra. As such, it is useful for the rational design of colloids and process variables for a broad range of photothermal applications.

The interest in colloidal plasmonic nanoparticles (NPs) has grown rapidly in recent years due in part to advances in particle synthesis and the emergence of applications that leverage the unique optical properties of the particles, especially localized surface plasmon resonance (LSPR)^1^,^2^,^3^,^4^,^5^,^6^,^7^,^8^. At LSPR there is a uniform and coherent oscillation of free electrons within the particles that gives rise to highly localized field enhancement and intense absorption of incident light. Moreover, the LSPR wavelength can be tuned from the ultraviolet (UV) through the near-infrared (NIR) spectrum by controlling the size, shape and structure the particle during synthesis^9^. The ability to tune LSPR in this fashion has proven useful for a variety of applications in fields that include biosensing, optical coherence tomography^10^, photoacoustic imaging^11^ and two-photon luminescence imaging^12^, among others. The main focus of the paper is on photothermal applications of plasmonic colloids wherein the absorbed optical energy at LSPR is used to heat the surrounding medium. Among the many applications of laser-induced photothermal heating, minimally-invasive cancer therapies have drawn particular interest due to their potential benefit and impact on human health. In such therapies, plasmonic (usually gold) nanoparticles are functionalized to enhance their biocompatibility and to enable targeting of malignant tissue. The particles are introduced into the vasculature where they circulate and preferentially bind to targeted tissue. They are then laser heated to a sufficiently high temperature to destroy the tissue. Various plasmonic nanoparticle structures have been studied for photothermal therapies including nanoshells^13^, nanorods^14^,^15^, nanotori^15^,^16^, and more recently nanocages^17^. Such particles can be designed from fist principles (shape and structure) to achieve LSPR at preferred NIR wavelengths that more effectively penetrate soft tissues for *in vivo* applications, as compared to other wavelengths^18^. The particle design can be accomplished using various theoretical methods and numerous groups have validated such predictions experimentally. However, few groups have considered the optimization of plasmonic colloids for photothermal applications subject to constraints on the particle concentration, which is the focus of this work.

In this paper, we introduce a computational approach to optimize the optical absorption of dilute monodisperse colloids of core-shell plasmonic NPs under the constraint of a prescribed volume fraction. Since the volume fraction is held constant, the particle concentration is size-dependent, i.e., colloids with larger particles have lower particle concentrations. We demonstrate the approach for monodisperse colloids of core-shell SiO_2_@Au and Fe_3_O_4_@Au nanoparticles with particle sizes and wavelengths that range from 5–100 nm and 600–1200 nm, respectively (allowing for the shell thickness to range from 0 to 100% of the particle radius). These colloids are chosen to illustrate important differences in optical absorption for particles with lossless (SiO_2_) vs. lossy (Fe_3_O_4_) dielectric core constituents. The optical absorption is predicted using Mie theory and the analysis shows that there is a unique mix of parameters (core radius, shell thickness, wavelength) for each of the two colloidal systems respectively, that maximize absorption, independent of the value of the volume fraction. Furthermore, we show that a carefully chosen lossy core produces a much broader absorption peak with much less sensitivity to variations in particle structure and wavelength as compared to a lossless core. This is important because it provides insight into the advantageous use of lossy dielectric particle constituents to tune colloidal absorption to compensate for variations in particle size etc. that can occur during synthesis. In should also be noted that this is one of very few studies that quantifies the optical absorption of Fe_3_O_4_@Au nanoparticles^19^. Finally, this approach is readily implemented and can be adapted to colloids of plasmonic nanoparticles with arbitrary shapes, structures and constituents using well-established numerical methods to compute optical absorption and scattering^20^,^21^. In fact, there are several commercial programs such as the Comsol Multiphysics software (www.comsol.com) that are widely used for such analysis. Specifically, the Comsol RF module is a finite-element based solver that can be used to predict the transient or time-harmonic optical response of nanoparticles with arbitrary shapes and constituents with user-defined lossy and dispersive dielectric functions^15^,^16^,^22^. As such, this approach presented here is useful for the rational design of dilute colloids and process variables for a broad range of photothermal applications.

## Results

We demonstrate the theory via application to dilute monodisperse colloids of SiO_2_@Au and Fe_3_O_4_@Au NPs subject to the constraint of a prescribed volume fraction. In this analysis, the particle separation is assumed to be sufficiently large (dilute) such that the photonic coupling between particles is negligible, i.e., the optical response of the colloid is essentially the same as the sum of the individual particles. The normalized absorption cross-section of the colloids, which is denoted by 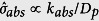, depends on the absorption efficiency *k*_*abs*_ as defined in Eqs (1, 2, 3), which is a function of the particle size, shell thickness and wavelength. It is important to note that since *ϕ* is held constant as we compare the absorption of colloids with different sized particles, the particle concentration is size-dependent, i.e. 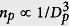. Thus the normalized absorption cross-section has a functional dependency of the form 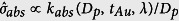 as in Eq. (14). We compare colloids of different sized particles in the range *D*_*p*_ ∈ [5, 100] nm (5 nm≤ *D*_*p*_≤ 100 nm) with shells that can ranges from 0 to 100% gold, i.e. *t*_*Au*_ ∈ [0, *D*_*p*_/2] nm, or equivalently *ξ*_*Au*_ ∈ [0, 100]. Note that if *t*_*Au*_ = *D*_*p*_/2 (*ξ*_*Au*_ = 100), the particle is a solid gold sphere. We allow for wavelengths in the range λ ∈ [600, 1200] nm. Mie theory was used to generate a 3D array of normalized absorption cross-sections 
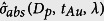
. Form this data we determine the mix of parameters (*D*_*p*_, *t*_*Au*_, *λ*) that produce the optimum normalized absorption cross-section, denoted 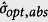.

Three distinct studies are performed to demonstrate the optimization process. In the first study, *D*_*p*_ and *λ* are varied over their respective ranges in 0.5 nm and 2 nm increments, respectively. For each pair (*D*_*p*_, *λ*) we determine the value of *ξ*_*Au*_ ∈ [0, 100] (i.e. the shell thickness) that produces the maximum absorption. We found that 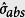 is a very sensitive function of *ξ*_*Au*_, and had to use very fine increments of this variable (*ξ*_*Au*_ = 0.001) to obtain smooth absorption data. This analysis results in a 2D array of optimized normalized cross-sections, which we denote by 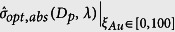. This data is presented as a surface plot over the *D*_*p*_ − *λ* plane. In the second study, we allow *λ* to vary freely over its range of values, and for each value, we determine the pair (*D*_*p*_, *ξ*_*Au*_) that maximize the absorption by searching *D*_*p*_ ∈ [5, 100] nm and *ξ*_*Au*_ ∈ [0, 100]. Thus, we obtain a 1D array, which is denoted by 
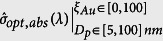
. Finally, in the third study, we allow *D*_*p*_ to vary over its range of values, and for each of these we determine the pair (*ξ*_*Au*_, *λ*) that maximize the absorption by searching the ranges *ξ*_*Au*_ ∈ [0, 100] and *λ* ∈ [600, 1200] nm. This produces a 1D array of optimized normalized cross-sections, which is denoted by 
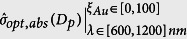
. Two plots are generated from this data. The fist plot shows 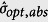 vs. *D*_*p*_. In the second plot, the optimum values of *ξ*_*Au*_ and *λ*, which are plotted as a function of *D*_*p*_ on the same graph. All of this data is presented for the two colloids separately in the following sections.

### SiO_2_@Au NP Colloids

**Figure 1:**
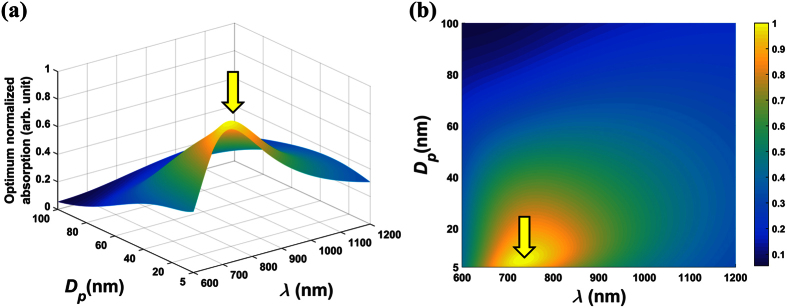
Optimum normalized absorption for SiO_2_-Au particles. (**a**) surface plot of 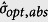. (**b**) 2D projection of surface plot.

**Figure 2:**
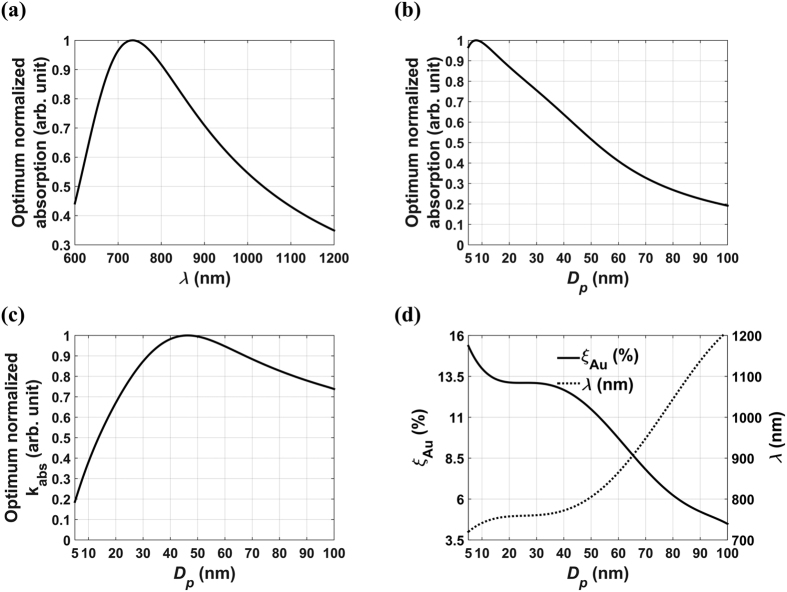
Optimum normalized absorption. (**a**) as a function of wavelength λ, (**b**) as a function of particle size *D*_*p*_, (**c**). optimum absorption efficiency *k*_*opt,abs*_ vs. particle size *D*_*p*_ and (**d**) optimum shell-to-radius ratio (solid line, left y-axis) and wavelength (dotted line, right y-axis).

The SiO_2_@Au nanoparticles have a lossless SiO_2_ core, i.e. 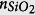 is real-valued as shown in Eq. (10). Therefore, all of the absorption is within the gold shell. The normalized absorption analysis for these colloids is shown in Figs 1 and 2, and the unnormalized peak absorption for these plots is 5.0962 × 10^8^m^−1^. Specifically, Fig. 1a,b show surface and projection plots of the optimized normalized absorption cross-section 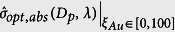, respectively. These plots show that the maximum absorption occurs for a particle diameter *D*_*p*_ = 8 nm with a shell thickness ratio *ξ*_*Au*_ = 14.43 (%) at a wavelength of *λ* = 734 nm (indicated by the arrow). These parameters define a global maximum over the entire range of independent variables (*D*_*p*_, *t*_*Au*_, *λ*). Moreover, since the volume fraction in this analysis was assumed to be constant, but otherwise arbitrary, these results are independent of a prescribed value of volume fraction and rather, apply to all monodisperse dilute colloids of SiO_2_@Au particles. It should be noted that in general, the optimum parameters depend on the allowed range of the independent variables. For example, if the incident wavelength were restricted to 800–1000 nm, the optimum particle size and shell thickness would be different. Figure 2 further illustrates the optimization. Figure 2a is a plot of the optimum normalized absorption as a function of wavelength, 
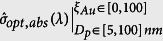
. The maximum absorption occurs at *λ* = 734 nm, which is consistent with Fig. 1a. A similar plot of absorption vs. particle size, 
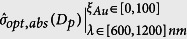
, is shown in Fig. 2b. Note that the absorption increases with decreasing particle size until it peaks at *D*_*p*_ = 8 nm, and then decreases thereafter. This can be understood from the relation 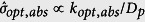 and an analysis of the optimum absorption efficiency *k*_*opt,abs*_, which is plotted as a function of *D*_*p*_ in Fig. 2c. A careful analysis of Fig. 2c shows that *k*_*opt,abs*_ increases faster than for *D*_*p*_ ∈ [5, 8] nm, increases slower than *D*_*p*_ for *D*_*p*_ ∈ [8, 46], and then decreases for larger particles, *D*_*p*_ > 46 nm. Thus, the profile shown in Fig. 2b is due to the behavior of *k*_*opt,abs*_ as shown in Fig. 2c. Finally, Fig. 2d shows the values of *ξ*_*Au*_ and *λ* that produce an optimum absorption for each particle size. Note that the relative shell thickness *ξ*_*Au*_ decreases with particle size while the wavelength redshifts. By cross-referencing Fig. 2b,d, we confirm that maximum absorption occurs when *D*_*p*_ = 8 nm, *ξ*_*Au*_ = 14.43 (%) and *λ* = 734 nm as indicated in Fig. 1.

### Fe_3_O_4_@Au NP Colloids

**Figure 3:**
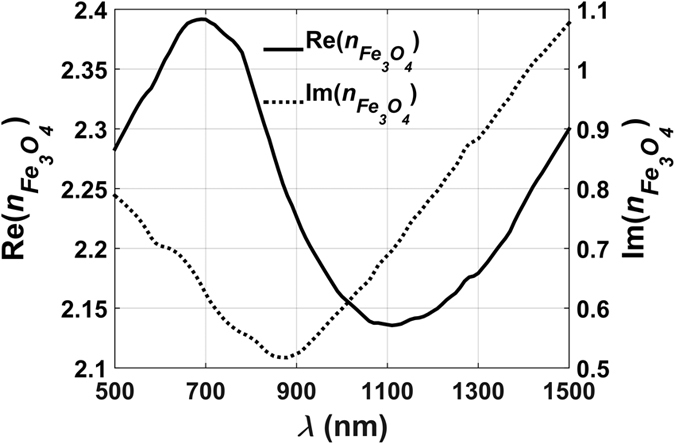
Refractive index of Fe_3_O_4_: real and imaginary components.

**Figure 4:**
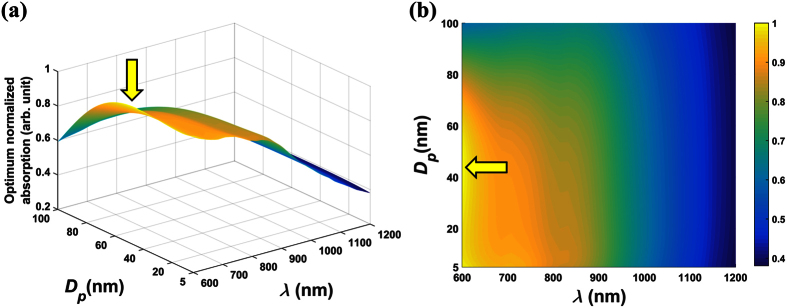
Optimum normalized absorption cross-section vs. *D*_*p*_ and *λ* for the Fe_3_O_4_@Au colloids (**a**). The surface plot and (**b**). the corresponding 2D projection of the surface plot.

**Figure 5:**
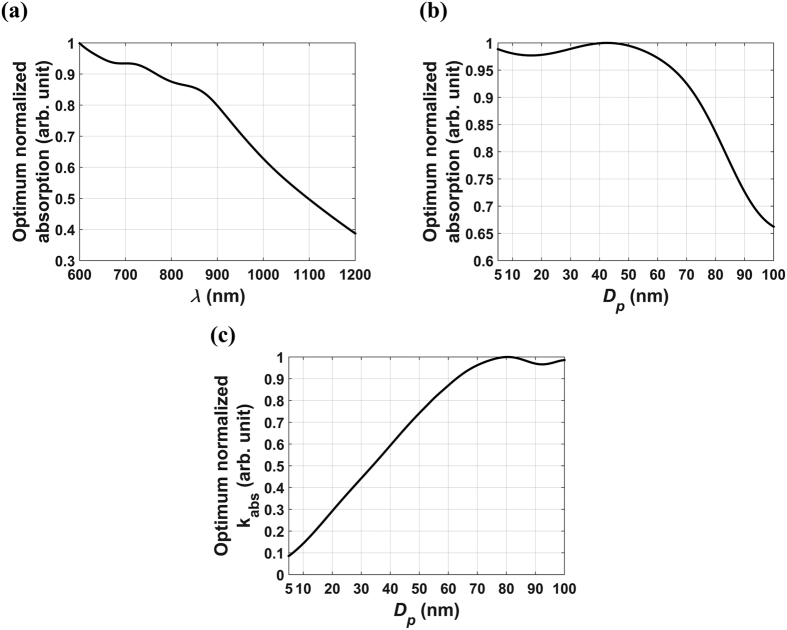
Optimum normalized absorption for Fe_3_O_4_@Au colloids. (**a**) Optimum absorption vs. *λ*, (**b**). optimum absorption vs. *D*_*p*_ and (**c**). optimum absorption efficiency *k*_*opt,abs*_ as a function of particle size.

We perform a similar analysis as above for the Fe_3_O_4_@Au Colloids. A major difference between these systems is that the Fe_3_O_4_ core is lossy, i.e. 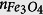 is complex-valued as shown in Fig. 3. Thus, the core and shell both contribute to absorption. The optimization normalized absorption analysis is shown in Figs 4 and 5, and the unnormalized peak absorption for these plots is 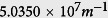. Figure 4a,b show surface and projection plots of the optimized absorption cross-section, respectively. The peak absorption occurs at *D*_*p*_ = 44 nm and *λ* = 600 nm as indicated by the arrow. Note that there is a relatively broad region of pronounced absorption that spans *D*_*p*_ ∈ [5, 70] nm and *λ* ∈ [600, 800] nm. In this region, the optimum normalized absorption is within 90% of its maximum value. This is also illustrated in Fig. 5a,b and is in contrast to SiO_2_@Au NP colloids that exhibit a relatively sharp and localized absorption peak as shown in Fig. 1b. The optimum absorption efficiency vs. particle size is plotted for comparison in Fig. 5c. It should be noted that our predictions are in excellent agreement with previous studies of Fe_3_O_4_@Au particles. For example, Chaffin *et. al.* predicted an LSPR wavelength of ~740 nm for a core-shell particle of *D*_*p*_ = 50 nm at *ξ*_*Au*_ = 10 (%)^23^. Our corresponding prediction is 750 nm and is based on a more rigous dielectric function for Fe_3_O_4_ as shown in Fig. 3.

**Figure 6:**
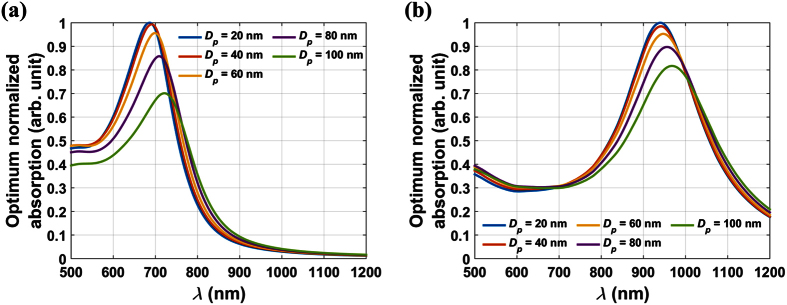
Parametric analysis of optimum normalized absorption vs. *λ* for different sized Fe_3_O_4_@Au NPs with a fixed shell-to-radius ratio *ξ*_*Au*_: (**a**). *ξ*_*Au*_ = 25(%), (**b**). *ξ*_*Au*_ = 10(%).

**Figure 7:**
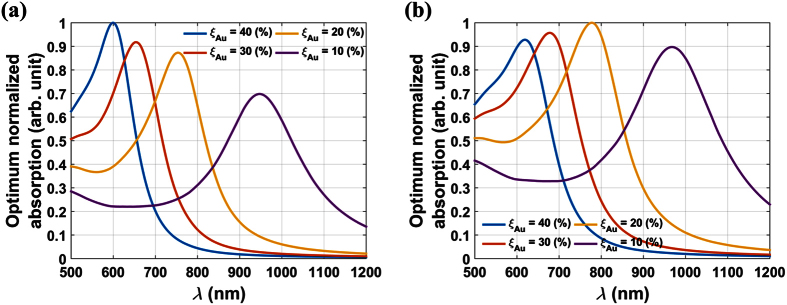
Parametric analysis of optimum normalized absorption vs. *λ* for two different sized Fe_3_O_4_@Au NPS with various shell-to-radius ratios *ξ*_*Au*_ (**a**). *D*_*p*_ = 60 nm, (**b**). *D*_*p*_ = 100 nm.

It is instructive to investigate the absorption spectrum of Fe_3_O_4_@Au NPs as a function of size and shell thickness. In Fig. 6a,b, we plot the optimum normalized absorption for different sized particles with the shell-to-radius ratio fixed for all particles at *ξ*_*Au*_ = 25(%) and *ξ*_*Au*_ = 10(%), respectively. These figures show that smaller particles have a higher absorption efficiency and a LSPR wavelength that redshifts as the particle size increases. In Fig. 7, we plot the optimum normalized absorption spectrum for two different sized particles *D*_*p*_ = 60 nm and 100 nm for a range of shell-to-radius ratios, *ξ*_*Au*_ = 10, 20, 30 and 40%. Note that the LSPR wavelength redshifts as this ratio decreases as expected. For the smaller particle (*D*_*p*_ = 60 nm), a thicker gold shell produces a higher absorption efficiency but at a lower LSPR wavelength. The highest absorption occurs for a solid gold particle at its LSPR wavelength (~537 nm). However, for the larger particle (*D*_*p*_ = 100 nm), there is less variation in absorption with shell thickness, and the highest absorption occurs at an intermediate thickness of *ξ*_*Au*_ = 20(%).

## Discussion

Colloids of plasmonic NPs of all types are under intense development for a broad range of applications in fields that span energy harvesting to healthcare^1^,^2^,^3^,^4^,^5^,^6^,^7^,^8^. These colloids with their unique optical properties hold potential as transformative agents for many applications, especially biomedical applications where they can enable enhanced and unprecedented multimodal biosensing, bioimaging and therapeutic functionality. While many theoretical and experimental studies of colloidal plasmonic NPs have been reported, relatively few have quantified photothermal optimization for dilute colloids subject to the constraint of a prescribed volume fraction. We have introduced a computational method that can be used to for this purpose and have demonstrated it for monodisperse SiO_2_@Au and Fe_3_O_4_@Au NP colloids. In our analysis, we have allowed the particle size and incident wavelength to range from 5 to 100 nm and 600–1200 nm, respectively and the shell thickness to range from 0 to 100% of the particle radius. We have found that there is a unique mix of parameters (core radius, shell thickness, wavelength), for each of the two respective colloidal systems, that optimize absorption. Moreover, these parameters depend on the allowed range of particle size and wavelength, but significantly, are independent of the prescribed volume fraction. The analysis also shows that the SiO_2_@Au colloids exhibit a much sharper absorption peak than the Fe_3_O_4_@Au colloids. This is due to the lossy Fe_3_O_4_ core in the latter that contributes significantly to absorption. This implies that a carefully chosen lossy core can be used to produce a much broader absorption peak with much less sensitivity to variations in particle structure and wavelength as compared to a lossless core. More broadly, lossy dielectric particle constituents can be introduced to tune colloidal absorption to compensate for variations in particle size etc. that can occur during synthesis. Finally, the method we present can be readily adapted to colloids of plasmonic NPs with arbitrary materials, shapes and structures by using appropriate numerical methods to compute the optical absorption cross-section. The approach should find considerable use for the rational design of colloids and process variables for a broad range of photothermal applications.

## Materials and Methods

### The Computational Model

We use Mie theory to predict the optical absorption of dilute colloids of core-shell plasmonic particles as a function of particle size and structure (core radius, shell thickness) and the incident wavelength. The extinction, scattering and absorption efficiencies *k*_*ext*_, *k*_*scat*_ and *k*_*abs*_ for the particles are computed using


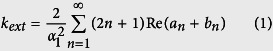



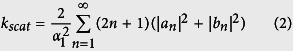



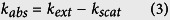


where *a*_*n*_ and *b*_*n*_ are scattering coefficients taken from Bohren and Huffman^24^, i.e.,


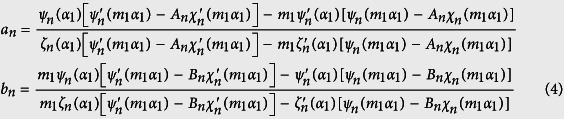


with


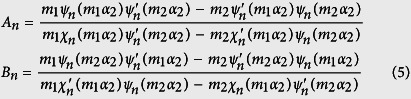


and


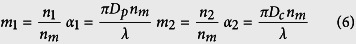


**Figure 8:**
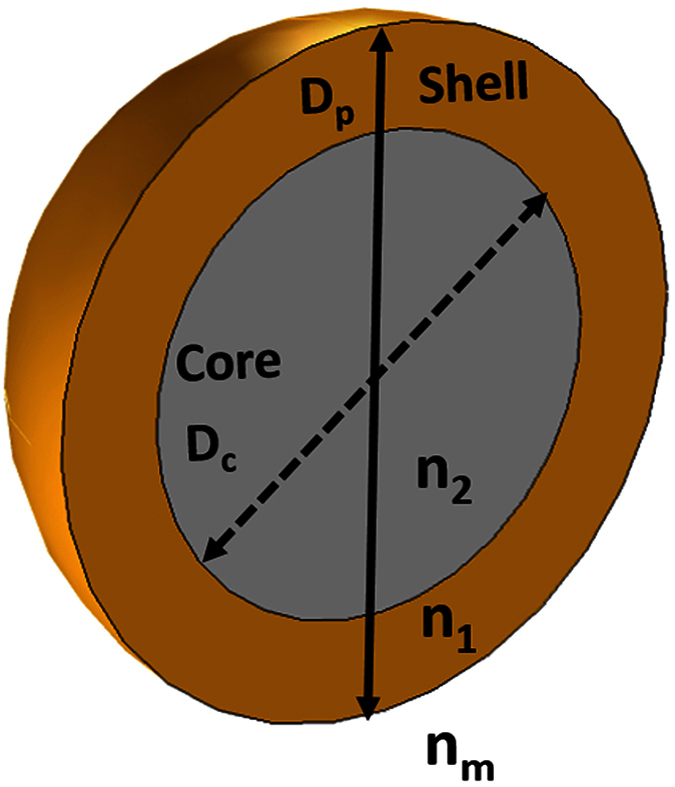
Core-shell particle structure and parameters.

**Figure 9:**
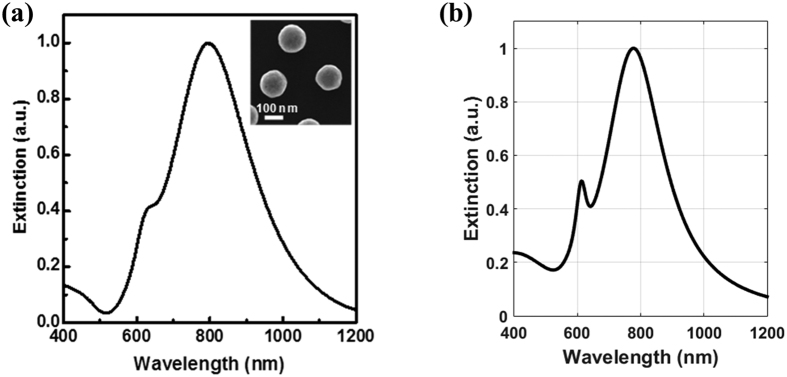
Mie theory model validation. (**a**) normalized experimental extinction data (adapted form ref. 25), and (**b**) theoretical extinction data.

Here *D*_*p*_ and *n*_1_ are the diameter and refractive index of the shell, respectively, *D*_*c*_ and *n*_2_ are the diameter and refractive index of the core and *n*_*m*_ is the refractive index of the surrounding medium (carrier fluid), which throughout this work is assumed to be H_2_O (Fig. 8). The shell has a thickness *t*_*Au*_ = (*D*_*p*_ − *D*_*c*_)/2, and we define a critical parameter *ξ*_*Au*_ ≡ (2*t*_*Au*_/*D*_*p*_) × 100 (%), which is the percentage of the shell thickness to the overall particle radius, i.e. *ξ*_*Au*_ = 100 represents a solid gold sphere. It is important to note that all materials in our study can be dispersive, i.e. *n*_1_, *n*_2_ and *n*_*m*_ can be complex-valued. The functions *ψ*_*n*_, *χ*_*n*_, *ζ*_*n*_ are Riccati–Bessel functions, which can be expressed as


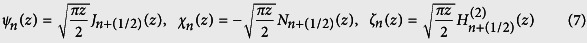


where *J*_*n*+(1/2)_, *N*_*n*+(1/2)_ represent, respectively, half integer-order Bessel functions of the first and second kind and 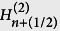 represents the half-integer-order Hankel function of the second kind. The extinction, scattering and absorption cross-sections of a particle, *σ*_*ext*_, *σ*_*scat*_ and *σ*_*abs*_, are just the product of the corresponding efficiency times the geometric cross-sectional area *A*_*p*_ e.g., 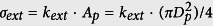 (m^2^), where *D*_*p*_ is the diameter of the particle. The comparison between our Mie theory-based model and corresponding experimental data is shown in Fig. 9. The particles in the experiment have a core radius of 62 nm (*D*_*c*_ = 124 nm), and a shell thickness of 14 nm (*t*_*Au*_ = 14 nm) as reported in the literature^25^. Note that there is excellent agreement between the theoretical and experimental extinction data.

### Material Properties

We apply Mie theory, Eqs (1, 2, 3, 4, 5, 6, 7), to dilute colloids of monodisperse (SiO_2_@Au) and (Fe_3_O_4_@Au) NPs. The synthesis of such particles has been described in the literature^19^,^26^. To model these particles, we need expressions for the refractive indices of the cores *n*_1_ = *n*_*SiO*2_ and 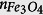, the shell, *n*_2_ = *n*_*Au*_ and the background medium, which is assumed to be water 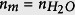. Moreover, we need to allow for the fact that the gold shells can be thinner than the mean free path of the free electrons (~42 nm). A dielectric function for gold that accounts for electron-surface scattering is ref. 27.


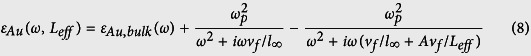


where *ε*_*Au*,*bulk*_ is the bulk dielectric function of gold, *ω* is the angular frequency of incident light, *ω*_*p*_ = 0.93 eV is the plasma frequency, *v*_*f*_ = 1.40 × 10^15^ nm/s is the Fermi velocity, l_∞_ = 42 nm is the mean free path of the free electrons, *A* is a dimensionless parameter, usually assumed to be close to unity (*A* = 1) and *L*_*eff*_ = *t*_*A*_ is the reduced effective mean free path of the free electrons (Fig. 8). The bulk dielectric function is given by an analytical expression that is based on an experiment-fitted critical points model^28^,^29^,^30^,


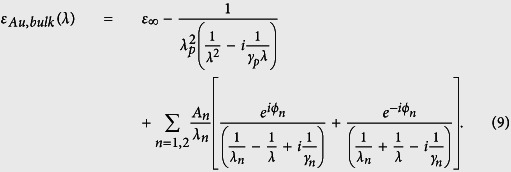


**Figure 10:**
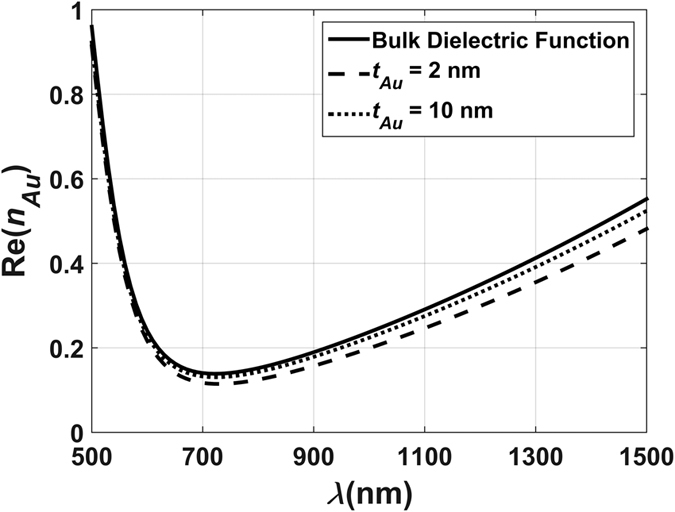
Real part of the refractive index of gold vs. shell thickness.

The parameters in Eq. (9) are defined in detail in the literature^28^,^29^,^30^. It is instructive to evaluate the impact of the electron-surface scattering term in Eq. (8). We find that it produces a slight deviation of the real part of *n*_*Au*_ from the corresponding bulk values at long wavelengths as shown in Fig. 10. There is virtually no difference in the imaginary part of *n*_*Au*_.

The SiO_2_ core is assumed to be lossless with a frequency-dependent refractive index of the form^27^


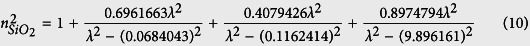


The refractive index of the surrounding medium, in this case H_2_O, is also assumed to be lossless i.e.,


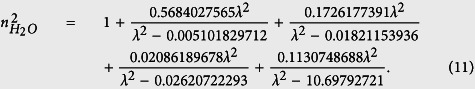


In these expressions *λ* is the vacuum wavelength in units of micrometers.

The Fe_3_O_4_ core is lossy and the refractive index of Fe_3_O_4_ at optical frequencies has not been widely reported. We obtain values for the real and imaginary components of 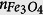 in tabular form by discretizing plots of measured data found in the literature^31^. The components used in our analysis are plotted in Fig. 3. As noted, since 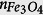 is complex-valued the core contributes to absorption in the Fe_3_O_4_@Au NPs.

### Optimization of Absorption

Our goal is to optimize the absorption of a colloid as a function of particle size, structure (core-shell dimensions) and wavelength subject to a prescribed volume fraction *ϕ*. Specifically, *ϕ* is held constant as we compare the absorption of monodisperse colloids with different-sized particles. The volume fraction is given by *ϕ* = *n*_*p*_*V*_*p*_, where *n*_*p*_ is the particle concentration (number per unit volume) and 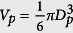 is the particle volume. If two different colloids, labeled i and j, have particle volumes then *n*_*p*,*i*_*V*_*p*,*i*_ = *n*_*p*,*j*_*V*_*p*,*j*_ = *ϕ*. Thus, *n*_*p*_ has a strong inverse dependency on particle size,


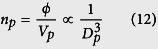


We assume that the colloid is dilute and that *ϕ* is sufficiently small (but otherwise arbitrary) so that photonic interactions between the particles can be neglected^32^. Thus, the extinction, scattering and absorption cross-sections of the colloid, *σ*_*col*,*ext*_, *σ*_*col*,*scat*_ and *σ*_*col*,*abs*_, can be modeled as the optical response of a single isolated immersed nanoparticle times the particle concentration^18^


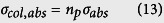


Combining Eqs (12 and 13) we find that,


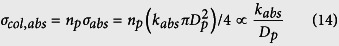


Thus, when the volume fraction is prescribed, the optimum absorption of a colloid depends on the ratio of the single particle absorption efficiency (k-factor) to the particle radius as in Eq. (14). In the remainder of this paper we simplify the notation and denote the absorption cross-section of the colloid by *σ*_*abs*_.

## Additional Information

**How to cite this article**: Xue, X. *et al.* Optimization of Optical Absorption of Colloids of SiO_2_@Au and Fe_3_O_4_@Au Nanoparticles with Constraints. *Sci. Rep.* **6**, 35911; doi: 10.1038/srep35911 (2016).

**Publisher’s note:** Springer Nature remains neutral with regard to jurisdictional claims in published maps and institutional affiliations.
